# Nanopore Sequencing Unveils Diverse Transcript Variants of the Epithelial Cell-Specific Transcription Factor Elf-3 in Human Malignancies

**DOI:** 10.3390/genes12060839

**Published:** 2021-05-29

**Authors:** Michaela A. Boti, Panagiotis G. Adamopoulos, Panagiotis Tsiakanikas, Andreas Scorilas

**Affiliations:** Department of Biochemistry and Molecular Biology, Faculty of Biology, National and Kapodistrian University of Athens, 15701 Athens, Greece; miboti@biol.uoa.gr (M.A.B.); ptsiak@biol.uoa.gr (P.T.); ascorilas@biol.uoa.gr (A.S.)

**Keywords:** alternative splicing, nanopore sequencing, transcription factor, long-read sequencing, transcriptome, ETS transcription factor 3 (Elf-3)

## Abstract

The human E74-like ETS transcription factor 3 (Elf-3) is an epithelium-specific member of the ETS family, all members of which are characterized by a highly conserved DNA-binding domain. Elf-3 plays a crucial role in epithelial cell differentiation by participating in morphogenesis and terminal differentiation of the murine small intestinal epithelium, and also acts as an indispensable regulator of mesenchymal to epithelial transition, underlying its significant involvement in development and in pathological states, such as cancer. Although previous research works have deciphered the functional role of Elf-3 in normal physiology as well as in tumorigenesis, the present study highlights for the first time the wide spectrum of *ELF3* mRNAs that are transcribed, providing an in-depth analysis of splicing events and exon/intron boundaries in a broad panel of human cell lines. The implementation of a versatile targeted nanopore sequencing approach led to the identification of 25 novel *ELF3* mRNA transcript variants (*ELF3* v.3–v.27) with new alternative splicing events, as well as two novel exons. Although the current study provides a qualitative transcriptional profile regarding *ELF3*, further studies must be conducted, so the biological function of all novel alternative transcript variants as well as the putative protein isoforms are elucidated.

## 1. Introduction

Alternative splicing (AS) constitutes a tightly regulated mechanism of eukaryotic cells that ensures their essential transcriptomic and proteomic diversity by producing multiple mRNA transcripts from a single gene [1]. AS mechanism is considered to contribute to cellular homeostasis by controlling a wide spectrum of physiological processes, such as cell cycle control, proliferation, apoptosis and angiogenesis [2]. During the process of AS, a megadalton machinery known as the spliceosome excises the intronic sequences of the precursor mRNAs (pre-mRNAs) and subsequently joins the exons together to generate mature mRNAs. Genome-wide studies have revealed that the overwhelming majority (~95%) of pre-mRNAs undergo AS, generating a covey of transcripts with differential structural and functional features [1,3]. The different combinations of exon splicing lead to the generation of distinct mRNAs from a single pre-mRNA, that may differ both in their untranslated regions (UTRs) and/or their coding sequence [4]. As a result, the produced alternative splice variants may exhibit differential protein-coding capacities, subcellular localizations, stability and functional roles [5]. In addition, AS has been proven to offer an additional layer of complexity regarding gene expression regulation, producing not only protein-coding mRNAs, but also non-coding RNAs, both of which can function as regulators of gene expression [6]. Therefore, deregulation of the splicing machinery has been linked to numerous human diseases, including cancer [7,8]. The development of malignant tumors is a multistep process, in which the genetic landscape of normal cells is modified, resulting in the acquisition of oncogenic characteristics [9]. Changes in the alternative splicing mechanism subserve the development of cancer-associated phenotypes by promoting angiogenesis [10], avoiding apoptosis [11] and inducing cell proliferation [12], invasion and metastasis [13,14].

In the last decade, the introduction of Next-Generation sequencing (NGS) has provided new insights into the transcriptome complexity and the research on AS, leading to the identification of previously unknown mRNA transcripts that conventional sequencing techniques were incapable of detecting [15,16,17]. However, even though NGS offered a tremendous sequencing capacity with unprecedented accuracy and depth, it still demonstrated serious limitations, with its most prominent disadvantage being the production of short reads (up to~600 bp), which hinders the efficient study of large structural variations in single reads, mainly by introducing bias during the assembly procedure [18,19]. These limitations were overcome with the newly introduced third-generation sequencing (TGS) methodology, such as the one offered by Oxford Nanopore Technologies (ONT). In contrast to NGS platforms that produce short reads, TGS is characterized by an improved sequencing chemistry, resulting in the generation of long reads, with an average length of more than 10 kb [20,21], therefore putting no limit on the length of DNA or RNA molecules that can be sequenced. The long-read technology of TGS is considered to be its most advantageous feature, since it drastically improved the analysis of genome structures and the quality of genome assembly [20,22,23]. Longer read lengths act as more representative elements of the total amount of genetic information and thus produce more contiguous reconstructions of the genome [24]. According to variation analysis studies, long reads paved the way for an easier identification of deletions, insertions, translocations and other structural changes that may exist [25]. Moreover, TGS can be successfully implemented for in-depth transcriptome research, having already resulted in the identification of novel transcript variants and gene fusions that would be really challenging to detect with NGS approaches [26]. Besides the higher error rates that are still observed, the recently introduced TGS technology is expected to facilitate the identification of novel mRNAs, deciphering the hallmark features of AS towards human pathophysiology.

One such example is the human E74-like ETS transcription factor 3 (Elf-3), which is an epithelium-specific member of the ETS family [27,28,29,30], whose members are characterized by a highly conserved DNA-binding domain, the ETS domain. The biological function of Elf-3 has been well documented during the implementation of transient transfection-reporter gene assays, suggesting that this protein carries out its biological effects by acting as a nuclear transcription factor [31,32,33,34,35,36]. In detail, previous studies have shown that Elf-3 plays a crucial role in the epithelial cell differentiation, not only by participating in morphogenesis and terminal differentiation of the murine small intestinal epithelium [37], but also by regulating the expression of keratinocyte [27,38,39], bronchial [40] and retinal [41] epithelial cell gene markers. Except for its essential cellular function in development, it has been recently revealed that Elf-3 acts as an indispensable regulator of mesenchymal to epithelial transition (MET), underlying its crucial role in epithelial state and implying its potential involvement in invasion and metastasis of cancer [42].

Other studies have directly implicated Elf-3 in the normal physiology of the breast, as well as in breast cancer [29,30,32,34,36,43,44]. Specifically, in the normal breast, *ELF3* is believed to be expressed in a subset of pluripotent ductal epithelial cells, which are retained after involution of the breast [36]. According to Schedin P. J. et al., stable *ELF3* expression leads to the transformation of MCF-12A human mammary epithelial cells, imparting to these cells a phenotype characterized by enhanced motility and invasiveness, as well as an epithelial-to-mesenchymal morphological transition (EMT) [45]. A few years after the evidence that this transcription factor plays an essential role in transformation of MCF-12A, it was documented that Elf-3 is capable of transforming MCF-12A via a second, novel, non-nuclear mechanism which involves the complicity of the cytoplasmic Elf-3. It was also demonstrated that the nuclear localization of Elf-3 protein induces apoptosis in non-transformed mammary epithelial cells via a transcription-dependent mechanism, giving Elf-3 an additional cellular function and strengthening its potential oncogenic role in human breast cancer [46].

Elf-3 is encoded by the human *ELF3* gene, which is located at chromosome 1(1q32.1). The pre-mRNA of *ELF3* is subjected to AS and therefore generates two alternative splice variants, *ELF3* v.1 and v.2 (GenBank^®^ accession numbers: NM_004433.5 and NM_001114309.2, respectively), which differ only in their 5′ UTR and encode a 42 kD protein of 371 aa [27]. This protein is characterized by the presence of five defined domains [47]. In particular, the N-terminal contains a pointed (PNT) domain, which has been implicated in protein-protein interaction and, thus, it is not considered to possess a functional role [27,42,48]. The next domain is a transactivation domain (TAD) that appears to contain an α-helix, and point mutations occurring in this α-helix disrupt Elf-3’s ability to activate promoter activity, as well as its interaction with the TATA box binding protein (TBP) [32,49]. It has also been documented that the apoptotic function of Elf-3 is expressed via TAD, underlying the critical contribution of the particular domain in Elf-3’s functionality [46]. A serine- and aspartic acid-rich (SAR) domain appears to influence cellular transformation when Elf-3 is localized to the cytoplasm, whereas a nuclear function for this domain has not been documented [30,46]. Unlike other ETS proteins, Elf-3 contains two AT-hook domains, which are involved in non-sequence-specific binding to AT-rich regions of DNA, as well as protein-protein interactions [47]. The AT-hook domain of Elf-3 also includes a bipartite nuclear localization signal (NLS), responsible for the transport of the protein inside the nuclear [46]. Finally, the ETS domain, which is structurally similar among the ETS members, is required for DNA-binding [27]. The multifaceted role of Elf-3 in regulation of gene expression, cell transformation and apoptosis as well as its poorly investigated transcriptional profile, prompted us to investigate the existence of novel *ELF3* transcripts, the identification of which could broaden our knowledge regarding Elf-3’s roles in eukaryotic cell and define its implications in pathological states, such as cancer.

## 2. Materials and Methods

### 2.1. Biological Material

For the implementation of the present study, a total of 52 human cell lines were propagated according to the American Type Culture Collection (ATCC) guidelines. In detail, the established panel of human cell lines was the following: OVCAR-3, SK-OV-3, ES-2, MDAH-2774 (ovarian cancer), Ishikawa, SK-UT-1B (endometrial adenocarcinoma), HeLa, SiHa (cervical carcinoma), MCF7, SK-BR-3, BT-20, MDA-MB-231, MDA-MB-468 (breast adenocarcinoma), BT-474, T-47D, ZR-75-1 (ductal carcinoma), PC-3, DU 145, LNCaP (prostate cancer), T24, RT4 (urinary bladder cancer), ACHN, 786-O, Caki-1 (renal cell carcinoma), Caco-2, DLD-1, HT-29, HCT 116, SW620, COLO 205, RKO (colorectal cancer), Hep G2, HuH-7 (hepatocellular carcinoma), AGS (gastric adenocarcinoma), A549 (lung adenocarcinoma), U-87 MG, U-251 MG, D54, H4, SH-SY5Y (brain cancer), FM3, MDA-MB-435S (melanoma), K-562, HL-60, Jurkat, REC-1, SU-DHL-1, GRANTA-519 (leukemia), Raji, Daudi, U-937 (lymphoma) and HEK-293 (normal embryonic kidney).

### 2.2. Total RNA Extraction and First-Strand cDNA Synthesis

Total RNA was isolated with the use of TRIzol™ Reagent (Ambion™, Thermo Fisher Scientific Inc., Waltham, MA, USA), following the guidelines of the manufacturer. All RNA samples were diluted in THE RNA Storage Solution (Ambion™), whereas their concentration and purity were assessed spectrophotometrically at 260 and 280 nm, using a BioSpec-nano Micro-volume UV-Vis Spectrophotometer (Shimadju, Kyoto, Japan).

In the next step, 2 μg of each RNA sample was used for the implementation of the first-strand cDNA synthesis. For this purpose, an oligo-dT_20_ was used as RT primer to anneal in the 3’ poly(A) tail of the mRNA molecules. Briefly, the cDNA synthesis was performed in reaction volumes of 20 μL, using 2 μg of total RNA, 1 μL of oligo-dT_20_ (10 μM), 1 μL dNTP Mix (10 mM each), 4 μL 5X First-Strand Buffer, 1 μL DTT (0.1 M), 1 μL (40 U) RNaseOUT™ (Invitrogen™, Thermo Fisher Scientific Inc., Waltham, MA, USA) and 1 μL (200 U) of SuperScript™ III Reverse Transcriptase (Invitrogen™, Thermo Fisher Scientific Inc., Waltham, MA, USA), according to the manufacturers’ protocol. For the quality control of the produced cDNA samples, the expression levels of the housekeeping gene Glyceraldehyde 3-phosphate dehydrogenase (*GAPDH*) were tested. Finally, equal amounts of each cDNA were pooled for the creation of a final cDNA mix from the 52 human cell lines.

### 2.3. PCR Amplification of ELF3 mRNA Transcripts

A touchdown PCR-based assay was carried out to amplify the mRNA transcript variants of the human *ELF3* gene with significantly increased sensitivity, specificity and PCR yield. In detail, a forward gene-specific primer (sequence: 5′-GCCAGATACCTCAGCGCTAC-3′) was designed to target the first annotated exon, and was used along with a reverse gene-specific primer (sequence: 5′-TCCGACTCTGGAGAACCTCT-3′) that was designed to anneal at the last annotated exon of *ELF3*.

The touchdown PCR was performed in reaction volumes of 25 μL, containing KAPA Taq Buffer A (Kapa Biosystems Inc., Wilmington, MA, USA), which includes MgCl_2_ at a final concentration of 1.5 mM, 0.2 mM dNTPs, 0.4 μM of each primer and 1 unit of KAPA Taq DNA Polymerase (Kapa Biosystems Inc.), in a Veriti 96-Well Fast Thermal Cycler (Applied Biosystems™). Furthermore, the applied cycling protocol that was followed included an initial denaturation step at 95 °C for 3 min, followed by 35 cycles of 95 °C for 30 s, 65 °C (auto-ΔTa: −0.3 °C/cycle) for 30 s, 72 °C for 2 min and a final extension step at 72 °C for 5 min. After the PCR run, the derived PCR product was purified with the NucleoSpin^®^ Gel and PCR Clean-up kit (Macherey-Nagel GmbH & Co. KG, Duren, Germany) before being used for the library construction.

### 2.4. Library Preparation and Nanopore Sequencing

A total of 1 μg purified PCR product was used as input for the targeted DNA-seq library preparation workflow. Nanopore sequencing was performed on a MinION Mk1C sequencer (Oxford Nanopore Technologies Ltd., Oxford, UK), using a FLO-MIN106D flow cell with R9.4.1 chemistry and the Ligation Sequencing Kit (SQK-LSK109, ONT), following the manufacturer’s protocol. In particular, the NEBNext^®^ Ultra™ II End Repair/dA-Tailing Module (New England Biolabs, Inc.) was used for the end repair process, the Agencourt AMPure XP beads magnetic beads (Beckman Coulter, Brea, CA, USA) were employed for the nucleic acid purification steps, while the adaptor ligation was accomplished with Quick T4 Ligase (New England Biolabs Inc., Ipswich, MA, USA) treatment.

### 2.5. Post Processing and Bioinformatics Analysis

The primary analysis of the acquired nanopore sequencing data, including base-calling, adapter trimming, as well as quality assessment, was performed with Guppy [50]. Nanopore sequencing reads were separated into two folders, “pass” and “fail”, based on their quality scores. Only the sequencing reads with quality score above a cut-off value, which are included in the “pass” folder, were used for the downstream analyses. The generated FASTQ files containing the raw sequencing data were aligned against the human reference genome (GRCh38), using the general-purpose Minimap2 aligner [51], whose parameters were adapted to perform spliced alignment. Alignment with Minimap2 led to the creation of a SAM output file, containing the sequencing reads that were successfully aligned against GRCh38. Then, the SAM file was converted into BAM, using samtools [52]. Mapped sequencing reads were visualized with the Integrative Genomics Viewer (IGV) software for the detection of the splice acceptor and donor sites and the existing UTRs [53]. Besides mapping with Minimap2, the detection of alternative splicing events in the created FASTQ file was also implemented with our developed algorithm “ASDT”, which was designed by members of our group as a generic splicing tool capable of identifying AS events and cryptic exons from high-throughput sequencing datasets.

### 2.6. Validation of the Novel Splice Junctions

The novel splicing events were validated with PCR-based assays, exploiting as template the cDNA pool that was used as the starting material for the sequencing. For this purpose, junction-specific primers were designed using the Primer-BLAST designing tool and used in specific combinations to target each novel splicing event (Tables S1 and S2).

## 3. Results

### 3.1. Nanopore Sequencing Reveals New Alternative Splicing Events of ELF3

**Figure 1:**
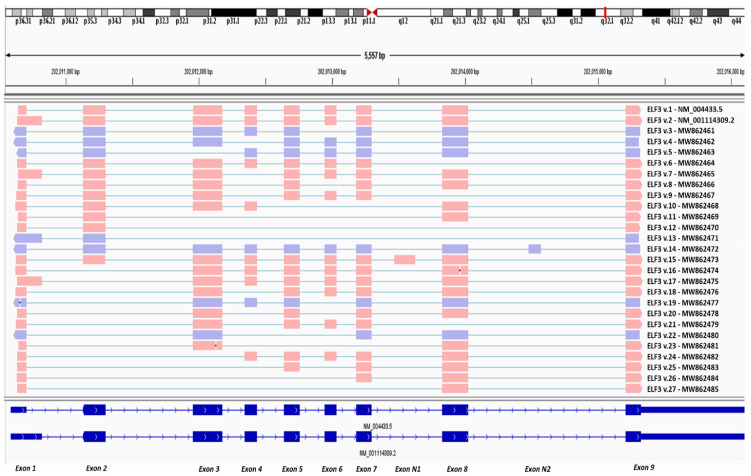
Visualization of the aligned sequencing reads representing novel alternative splice variants of the human *ELF3* gene, using Integrative Genomics Viewer (IGV).

Both the computational analysis with our algorithm ASDT as well as the visualization of the successfully aligned reads with IGV, confirmed the existence of all annotated splice junctions that are present in the annotated *ELF3* protein-coding transcripts v.1 and v.2 (GenBank^®^ accession numbers: NM_004433.5 and NM_001114309.2, respectively). Besides the detection of the annotated *ELF3* transcripts however, the computational analysis also led to the identification of novel, less abundant *ELF3* mRNA transcripts, which contain new alternative splicing events, as well as two novel exons. The sequencing reads that correspond to the novel transcripts were successfully aligned against the reference genome GRCh38, as observed in IGV (Figure 1). These novel findings were also identified, and thus validated, with the usage of our algorithm “ASDT”, which additionally provides the raw sequencing reads that correspond to each finding (Figures S1 and S2). Overall, the present study led to the identification of twenty-five novel *ELF3* transcripts (*ELF3* v.3–v.27), whose nucleotide sequences were deposited in GenBank^®^ (GenBank ID: MW862461, MW862462, MW862463, MW862464, MW862465, MW862466, MW862467, MW862468, MW862469, MW862470, MW862471, MW862472, MW862473, MW862474, MW862475, MW862476, MW862477, MW862478, MW862479, MW862480, MW862481, MW862482, MW862483, MW862484, MW862485).

Interestingly, a total of 15 novel alternative splicing events between annotated exons of *ELF3* were confirmed with significant sequencing depth. In detail, bioinformatics analysis revealed the existence of the previously unknown splicing events between exon 1(120 bp) and 3, as well as the alternative exon 1 (234 bp) and 3, both of them deriving from exon skipping of exon 2 (Table 1). Additionally, while exon 1 (120 bp) was found to be alternatively spliced with exons 3, 4, 5, 7 and 8, the alternative exon 1 (234 bp) was found to be spliced only with exon 3.

Besides the new findings that involve new splicing events of the first exons (120 bp and 234 bp), our results confirmed the splicing event between exons 3 and 5, which derives from exon skipping of exon 4. In addition, exon 3 is alternatively spliced with exons 7 and 8, two novel splice junctions that derive from multiple cassette exons. Furthermore, our findings confirmed that exon 2 is alternatively spliced not only with exon 4, but also with exon 8 and with the last exon of the gene (exon 9). In addition, two more novel splicing events that involve exon 7 were identified. Specifically, exon 7 was found to be alternatively spliced with exon 5 and exon 9, generating two novel splice junctions, which derive from exon skipping of exon 6 and exon 8, respectively. Finally, the last novel splicing event between annotated exons of *ELF3* corresponds to the exon skipping of exons 5, 6 and 7, and it is produced by the splicing of exon 4 with exon 8 (Table 1).

### 3.2. Detection of Two Novel Cryptic Exons of ELF3

**Figure 2:**
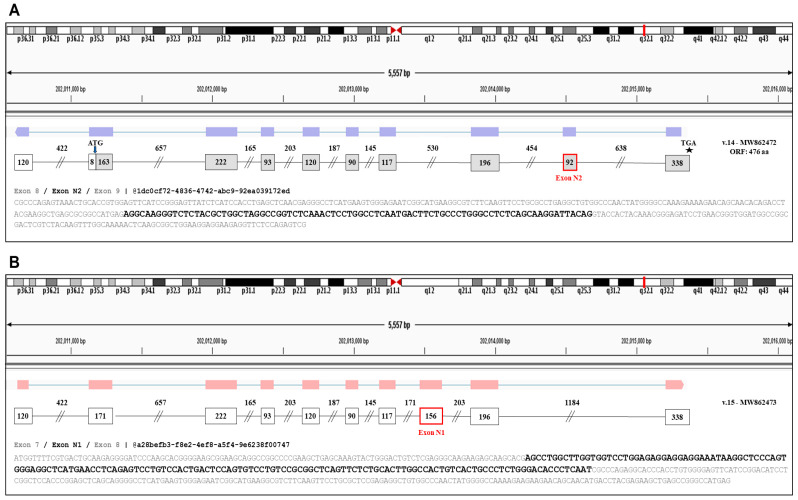
Schematic demonstration of the two identified exons (N1 and N2) as well as indicative sequencing reads verifying them. (**A**) Alignment of the new transcript *ELF3* v.14 that contains Exon N2. Based on the nanopore sequencing data, exon N2 is located between the annotated exons 8 and 9. (**B**) Alignment of the new transcript *ELF3* v.15 that contains Exon N1. Based on the nanopore sequencing data, exon N1 is located between the annotated exons 7 and 8.

Furthermore, analysis of the nanopore sequencing dataset led to the identification of two novel exons, named N1 and N2, which were aligned by numerous sequencing reads (Figure 2). Based on the raw sequencing data, the identified exon N1 is located between the annotated exons 7 and 8, having a length of 156 nt, whereas exon N2 is located between the annotated exons 8 and 9, and has a total length of 92 nt.

### 3.3. Identification of Novel ELF3 Transcripts Sharing the Annotated Initiation Codon

**Figure 3:**
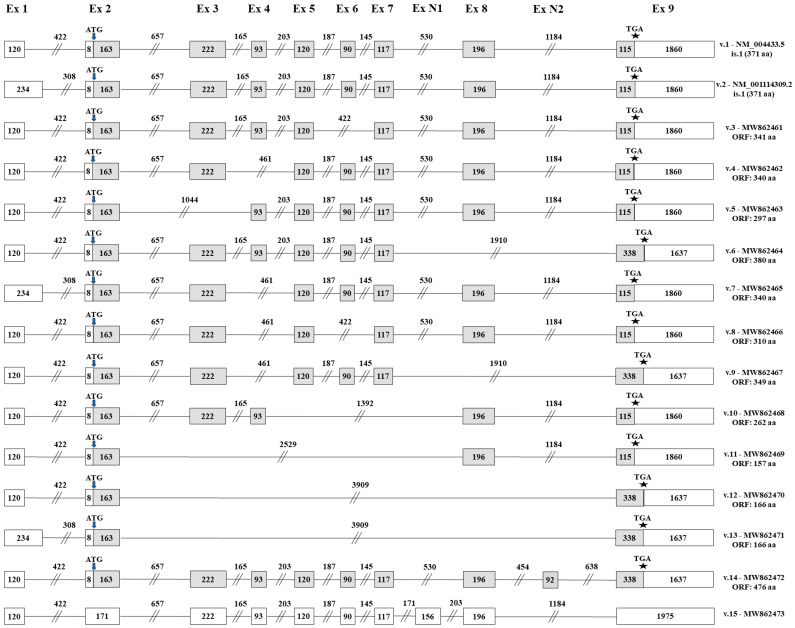
Detailed structure of the novel *ELF3* transcript variants sharing the annotated initiation codon. Exons are shown as boxes and introns as lines, while the numbers that characterize every box and line indicate their length in nucleotides. Gray and white boxes represent coding and non-coding exons, respectively. The positions of the ATG and TGA codons are pointed out with arrows (↓) and asterisks (*), accordingly.

TGS confirmed the existence of eleven novel *ELF3* transcripts (*ELF3* v.3–v.13) that are produced by differential splicing combinations between the annotated exons and yet retain exon 2, where the annotated initiation start codon resides (Figure 3). Of note, besides *ELF3* v.7 and v.13 that are characterized by the alternative exon 1 (234 bp), the rest of the novel transcripts encompass exon 1 (120 bp) that exists in the sequence of the main *ELF3* transcript (NM_004433.5). Additionally, open reading frame (ORF) was queried in the nucleotide sequence of each splice variant to predict their protein-coding capacity. Analysis showed that all eleven novel *ELF3* transcripts have ORFs, utilizing the annotated initiation start codon, and therefore they are all predicted to encode novel Elf-3 isoforms.

Based on the raw sequencing data, these novel transcripts are differentiated by several exon skipping events downstream of exon 2. Specifically, the four novel transcripts *ELF3* v.3–v.6 are derived by the exon skipping of exons 6, 4, 3 and 8, respectively (Figure 3). Furthermore, *ELF3* v.7–v.9 completely lack the annotated exon 4, thus containing the new splicing event between exons 3 and 5. Transcript *ELF3* v.7 is completely identical with *ELF3* v.4 downstream of exon 2, but it is differentiated since it contains the alternative exon 1 (234 bp). Regarding *ELF3* v.8 and v.9, besides the exon skipping of exon 4, they contain additional exon skipping events (Figure 3). Nanopore sequencing also revealed four novel *ELF3* transcripts (*ELF3* v.10–v.13), which lack at least three annotated exons and as a result they are characterized by significantly truncated cDNA sequences as compared to the main *ELF3* transcript.

Besides the aforementioned novel transcripts that are produced by new splicing events between already known exons, bioinformatics analysis led to the identification of two novel *ELF3* transcript variants (*ELF3* v.14 and v.15), which are characterized by the existence of novel exons in their cDNA sequences. Briefly, the novel exon N2 is present in the newly identified transcript *ELF3* v.14, while the novel exon N1 exists in the new transcript *ELF3* v.15 (Figure 3). It should be mentioned that although *ELF3* v.14 and v.15 contain new exons in their nucleotide sequences, they are not characterized by any exon skipping and therefore contain all the annotated exons that are present on the main transcript *ELF3* v.1. Finally, ORF query showed that *ELF3* v.14 has an ORF of 476 aa and thus is predicted to encode a novel protein isoform, whereas *ELF3* v.15 contains a premature termination codon and is most likely a non-coding RNA transcript.

### 3.4. Identification of Novel ELF3 Transcripts Bearing Novel Translation Initiation Codons

**Figure 4:**
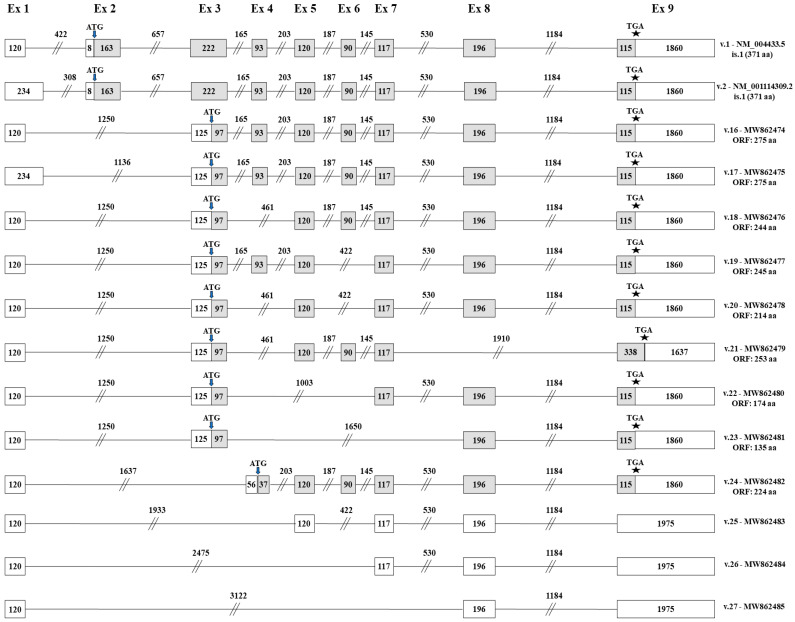
Detailed structure of the novel *ELF3* transcript variants bearing alternative translation initiation codons. Exons are shown as boxes and introns as lines, while the numbers that characterize every box and line indicate their length in nucleotides. Gray and white boxes represent coding and non-coding exons, respectively. The positions of the ATG and TGA codons are pointed out with arrows (↓) and asterisks (*), accordingly.

The obtained results also unveiled the existence of 12 *ELF3* transcripts (*ELF3* v.16–v.27), which completely lack the sequence of exon 2 and consequently the annotated translation initiation codon (Figure 4). From these transcripts, *ELF3* v.16–v.23 share the novel splicing event between exon 1 and exon 3. Briefly, both *ELF3* v.16 and v.17 lack exon 2, but are differentiated since *ELF3* v.16 contains the exon 1 (120 bp), while *ELF3* v.17 has the alternative exon 1 (234 bp). In addition, *ELF3* v.18 lacks exon 4, while *ELF3* v.19 lacks exon 6. The rest four *ELF3* transcripts (*ELF3* v.20–v.23) present notably truncated nucleotide sequences as compared to the annotated *ELF3* v.1, since they contain multiple exon skipping events, thus lacking at least three annotated exons (Figure 4). An ORF query in these transcripts indicated that *ELF3* v.16–v.23 have ORFs, utilizing an alternative initiation codon residing at exon 3, thus being predicted to encode novel protein isoforms. Finally, raw sequencing data revealed four novel transcripts (*ELF3* v.24–v.27) that lack both exons 2 and 3 and at the same time they contain novel alternative splicing events between distant exons of the gene. From these variants, only *ELF3* v.24 is predicted to have ORF, utilizing an alternative initiation codon located in exon 4 (Figure 4).

## 4. Discussion

In the current work, we used nanopore sequencing to identify new alternative splicing events of the human *ELF3* gene. Notably, despite our research mainly being focused on the detection of novel splicing events occurring during RNA processing, the identification of the presented 25 novel *ELF3* transcripts was implemented by targeted DNA-seq, instead of using the direct RNA sequencing application [54]. The selected approach resulted in a tremendous sequencing depth regarding *ELF3*, compared to conventional NGS and TGS applications, thus allowing the thorough identification of previously unknown low-frequent mRNA transcripts in single reads (Figure S3).

To the best of our knowledge there is a limited number of studies examining the signaling cascades in which Elf-3 is involved. Most of the studies highlight its significant role in epithelial cell differentiation predominantly by repressing the early-differentiation genes, like keratin 4, and activating late-differentiation markers, such as SPR2A. Moreover, regarding its involvement in MET, it was recently found that Elf-3 activates the expression of Grhl3 by binding onto its promoter. Subsequently, GRHL3 transcription factor, which plays a fundamental role during the initiation of MET, activates the expression of E-Cadherin, a key molecule for the epithelial state (Figure S4) [42,55].

The human *ELF3* gene displays a notable upregulation in human malignancies, associated with poor survival outcomes in HER2+ breast cancer patients [56] and poor diagnosis in patients with papillary thyroid cancer [57]. All the annotated *ELF3* transcript variants encode a 42 kD protein of 371 aa, which is structurally characterized by the presence of five domains. Each domain is responsible for a distinct Elf-3’s function, including transcriptional activation, apoptosis or cell transformation [47]. Consequently, the identification of the presented novel *ELF3* transcripts that may encode new Elf-3 isoforms encompassing a subset of these domains, could furnish new insights regarding the involvement of *ELF3* in physiological, as well as pathological, conditions. In fact, based on the *in-silico* analysis that was performed for the potential existence of ORFs in the cDNA sequences of *ELF3* v.3–v.27, 21 of them are predicted to encode new protein isoforms, whereas the remaining four transcript variants contain PTCs, thus being candidates for nonsense-mediated mRNA decay (NMD).

Transcript variants v.3, v.4, v.5, v.7, v.8, v.10 and v.11 have ORFs and at the same time they share the annotated start and stop codons. *ELF3* v.3 is predicted to encode a protein isoform of 341 aa, which includes all the domains of Elf-3, apart from SAR. Therefore, the potential translation of *ELF3* v.3 would lead to the generation of a novel protein that would retain the apoptotic and transcription factor functionalities, but would fail to mediate cell transformation from the cytoplasm [32,46,49]. *ELF3* v.4 and v.7 are predicted to encode the same protein isoform, which is a protein of 340 aa. These transcript variants contain all the exons that are necessary for the formation of the PNT, A/T hook, SAR and ETS domains, but they lack exon 4, which is crucial for the formation of an intact TAD (Table 2). Consequently, the corresponding novel protein isoform is predicted to be unable to mediate apoptosis and transcriptional activation [32,46,49]).

*ELF3* v.5 is predicted to encode a protein isoform of 297 aa that lacks only PNT domain. This domain is not critical for the transcription activity of Elf-3 or its apoptotic function and, thus, the existence of the rest four domains in the novel protein isoform suggests the synthesis of a molecule similar to the annotated Elf-3 [27]. The transcript variants *ELF3* v.8 and v.10 are predicted to encode novel protein isoforms of 310 aa and 262 aa, respectively. Both proteins lack an SAR domain and, therefore, they would not be capable of mediating cell transformation via the known cytoplasmic mechanism [46]. However, the protein isoform that is predicted to be encoded by *ELF3* v.10 contains TAD, but the absence of A/T-hook domain does not enable the transport of the particular protein into the nuclear, thus its involvement in apoptosis and transcriptional activation is not likely [47]. On the contrary, *ELF3* v.11 is predicted to encode a significantly truncated protein of 157 aa, which contains none of the five domains that characterize Elf-3. As a result, this protein isoform is less likely to be functional.

The other five *ELF3* transcript variants that include exon 2 utilize an alternative translation stop codon located downstream of the canonical one (v.6, v.9, v.12, v.13 and v.14). *ELF3* v.6 is predicted to encode a novel isoform of 380 aa, which includes TAD, SAR and A/T-hook domains, thus being able to function as a transcription factor and to mediate apoptosis (Table 2). Furthermore, *ELF3* v.9 is predicted to encode an isoform that contains SAR, A/T-hook and ETS domains. The existence of A/T-hook domain indicates the transport of this isoform in the nucleus, but no assumption about its nuclear function can be made. On the contrary, *ELF3* v.12 and v.13, which are the most truncated transcript variants that were identified during the present study, are predicted to encode the same protein isoform that consists of 166 aa. This isoform lacks all the domains that are present in the annotated Elf-3 and therefore it most likely represents a non-functional protein. Regarding *ELF3* v.14 that contains the novel exon N2, it is predicted to encode an isoform of 476 aa, which contains most of the crucial domains of the annotated Elf-3 protein (Table 2). In particular, the insertion of N2 interrupts the continuity of the ETS domain, leading to the generation of a novel Elf-3 isoform with reduced capability in terms of DNA-binding.

Additionally, nine novel transcript variants lacking exon 2 were found to contain ORFs utilizing an alternative translation initiation codon (*ELF3* v.16–v.24). In detail, *ELF3* v.16 and v.17 are predicted to encode the same protein isoform, which contains TAD, SAR, A/T-hook and ETS domains and, as a result, is expected to exhibit the same functionality with the annotated Elf-3 (Table 2). Furthermore, *ELF3* v.18, v.21 and v.24 are predicted to encode new protein isoforms that lack TAD domain, which is critical for the transcriptional and apoptotic function of nuclear localized Elf-3, but include the SAR domain and thus, they would be able to mediate cellular transformation when localized in cytoplasm. In addition, the isoform encoded by *ELF3* v.21 is characterized by an extended C-terminal, since the translational stop codon is located downstream of the annotated one (Figure 4). Another promising transcript is *ELF3* v.19. This variant is predicted to encode a new protein isoform with TAD, A/T-hook and ETS domains. The existence of these three critical domains suggests the synthesis of a protein isoform that retains Elf-3’s apoptotic function. On the contrary, *ELF3* v.20 and v.22 are predicted to encode new protein isoforms, which lack both TAD and SAR domains and therefore they are not expected to have any of the Elf-3’s functional properties. The last transcript variant that contains ORF, *ELF3* v.23, is predicted to encode a significantly truncated protein that includes only the ETS domain, hence it most likely lacks any biological functionality [27].

## 5. Conclusions

In conclusion, our study highlights for the first time the wide spectrum of previously unknown *ELF3* mRNAs that are transcribed, providing an in-depth overview in a broad panel of human cell lines. A total of 25 novel *ELF3* mRNA transcript variants (*ELF3* v.3–v.27) were identified, by designing and implementing a versatile targeted nanopore sequencing approach. Based on the identified exon/intron boundaries, 21 of the novel *ELF3* transcript variants contain ORFs, and thus are highly expected to encode new Elf-3 protein isoforms. The rest of the alternative splicing variants contain PTCs and represent NMD candidates. Although the current study provided a qualitative transcriptional profile regarding *ELF3,* further studies must be conducted, so the biological function of all novel, alternative transcript variants, as well as the putative protein isoforms, is elucidated.

## Figures and Tables

**Table 1:** List of annotated and novel splice junctions that were detected, as well as the number of nanopore sequencing reads covering, thus verifying each junction.

	Splice Junction between Known Exons	Nanopore Sequencing Reads Confirming Each Splice Site
Annotated	*Exon 1 (120 bp)–Exon 2*	205.399
	*Exon 1 (234 bp)–Exon 2*	3.328
	*Exon 2–Exon 3*	206.117
	*Exon 3–Exon 4*	210.848
	*Exon 4–Exon 5*	188.589
	*Exon 5–Exon 6*	221.945
	*Exon 6–Exon 7*	218.907
	*Exon 7–Exon 8*	213.796
	*Exon 8–Exon 9*	207.222
Novel	*Exon 1 (120 bp)–Exon 3*	11.469
	*Exon 1 (120 bp)–Exon 4*	2.138
	*Exon 1 (120 bp)–Exon 5*	5.321
	*Exon 1 (120 bp)–Exon 7*	1.114
	*Exon 1 (120 bp)–Exon 8*	1.627
	*Exon 1 (234 bp)–Exon 3*	22.197
	*Exon 2–Exon 4*	1.274
	*Exon 2–Exon 8*	6.339
	*Exon 2–Exon 9*	1.828
	*Exon 3–Exon 5*	4.211
	*Exon 3–Exon 7*	828
	*Exon 3–Exon 8*	8.047
	*Exon 4–Exon 8*	4.896
	*Exon 5–Exon 7*	140.394
	*Exon 7–Exon 9*	1.495

**Table 2:** Domains of the annotated Elf-3 protein and their presence in the novel protein isoforms that are predicted to be encoded by the newly identified *ELF3* transcripts.

Novel Transcripts	Elf-3 Domains				
	PNT	TAD	SAR	A/T	ETS
v.3	✓	✓	-	✓	✓
v.4	✓	-	✓	✓	✓
v.5	-	✓	✓	✓	✓
v.6	✓	✓	✓	✓	-
v.7	✓	-	✓	✓	✓
v.8	✓	-	-	✓	✓
v.9	✓	-	✓	✓	-
v.10	✓	✓	-	-	✓
v.11	-	-	-	-	✓
v.12	-	-	-	-	-
v.13	-	-	-	-	-
v.14	✓	✓	✓	✓	-
v.16	-	✓	✓	✓	✓
v.17	-	✓	✓	✓	✓
v.18	-	-	✓	✓	✓
v.19	-	✓	-	✓	✓
v.20	-	-	-	✓	✓
v.21	-	-	✓	✓	-
v.22	-	-	-	✓	✓
v.23	-	-	-	-	✓
v.24	-	-	✓	✓	✓

## Data Availability

The novel nucleic acid sequences presented in the current study, which correspond to the novel *ELF3* cDNA sequences (*ELF3* v.3–v.27), have been submitted and deposited to the GenBank^®^ Data Library, under the accession numbers MW862461–MW862485, accordingly.

## Supplementary Materials

The following are available online at https://www.mdpi.com/article/10.3390/genes12060839/s1, Table S1: Primers used in RT-PCR for the validation of the novel splice junctions. The number(s) appearing in primer names denote the number of each exon, while “/” shows that the primer is designed to target a particular splice junction; “N” indicates a novel exon, and “alt” stands for “alternative exon”. Melting temperature (T_m_) was calculated by Primer-BLAST. Table S2: Primer pairs used in RT-PCR for the validation of each novel splice junction. Figure S1: Nanopore sequencing reads verifying the presented novel transcripts *ELF3* v.3–v.15. Figure S2: Nanopore sequencing reads verifying the presented novel transcripts *ELF3* v.16–v.27. Figure S3: Alignment of publicly available sequencing reads to the reference sequence of *ELF3*. (a) NGS short-read datasets derived from the human Hep G2 cell line, validating the existence of two novel splicing events. (b) TGS long-read datasets derived from the HCT 116 cell line, validating the existence of splice junctions between exons 1 and 3 as well as 2 and 4, which are described in the present study. (c) TGS long-read datasets derived from the human HCT 116 cell line, validating the existence of the described novel exons (N1 and N2). Figure S4: Elf-3 acts as a key regulator during (a) epithelial cell differentiation and (b) mesenchymal to epithelial transition (MET).
