# Supplementary material for: Nanopore Sequencing Unveils Diverse Transcript Variants of the Epithelial Cell-Specific Transcription Factor Elf-3 in Human Malignancies
Source: Genes (Basel). 2021 May 29;12(6):839. doi: 10.3390/genes12060839 (PMC8227732; doi:10.3390/genes12060839)
Supplement: Supplementary file 1 [file genes-12-00839-s001.zip › Supplementary Table 1.pdf]

Supplementary Table S1. Primers used in RT-PCR for the validation of the novel splice junctions. The number(s) appearing in primer names denote the number of each exon, while "/" shows that the primer is designed to target a particular splice junction; "N" indicates a novel exon, and "alt" stands for "alternative exon". Melting temperature (T_m) was calculated by Primer-BLAST.

Direction	Name	Sequence (5′→3′)	Length (nt)	T_m (°C)
Forward	1/3F	CTCCGCCACTCCGAGAAG	18	59.5
	1alt/3F	TCCGAGCAAGAGCAGAAGG	19	59.4
	1/4F	CGCCACTCCGCTTCCAG	17	60.5
	1/5F	CCGCCACTCCGACCAGG	17	62.2
	1/7F	CGCCACTCCGATGGTTTTCG	20	62.0
	1/8F	CGCCACTCCGCGCCCAG	17	66.4
	2/4F	ATTGGAGGGTACAGCTTCCAGC	22	62.3
	2/5F	CATTGGAGGGTACAGACCAGG	21	59.8
	2/8F	GGAGGGTACAGCGCCCAG	18	62.2
	2/9F	GTCATTGGAGGGTACAGGTACTA	23	58.7
	3/5F	GAGACCTCAACCAGGGCAG	19	59.7
	3/7F	CTGCGAGACCTCAATGGTTTTC	22	59.8
	3/8F	CGAGACCTCACGCCCAG	17	59.5
	3/9F	TGCGAGACCTCAGTACTACTACA	23	60.1
	4/8F	AGGGCCCTTTGCGCCCA	17	64.6
	4/9F	CAGGGCCCTTTGGTACTACTA	21	58.5
	5/7F	TCTCCACCGCAGATGGTTTTC	21	60.6
	5/9F	GTCTCCACCGCAGGTACTACTA	22	60.7
	6/9F	CTTCCCCAGCGGTACTACTAC	21	59.3
	7/9F	GAAGAGCAAGCACGGTACTACTA	23	59.9
	N1/8F	ACACCCTCAATCGCCCAGAG	20	61.9
	8/N2F	GCCATGAGAGGCAAGGGTC	19	60.5
Reverse	3R	CTCAAGGGCACAATTGCAGAG	21	59.8
	5R	GTGGAGACGTCAGAGCTGC	19	60.5
	7R	GCTTGCTCTTCTTGCCCTC	19	58.8
	8R	GGCTCAGCTTCTCGTAGGTC	20	59.9
	9R	TCCGACTCTGGAGAACCTCT	20	59.3
