# Supplementary material for: Nanopore Sequencing Unveils Diverse Transcript Variants of the Epithelial Cell-Specific Transcription Factor Elf-3 in Human Malignancies
Source: Genes (Basel). 2021 May 29;12(6):839. doi: 10.3390/genes12060839 (PMC8227732; doi:10.3390/genes12060839)
Supplement: Supplementary file 1 [file genes-12-00839-s001.zip › Supplementary Table 2.pdf]

Supplementary Table S2. Primer pairs used in RT-PCR for the validation of each novel splice junction.

**Splice junction**	**Primer name**		**Amplicon size (bp)**
	**Forward**	**Reverse**	
Exon 1 (120 bp) - Exon 3	1/3F	3R	174
Exon 1 (234 bp) – Exon 3	1alt/3F	3R	174
Exon 1 (120 bp) - Exon 4	1/4F	5R	162
Exon 1 (120 bp) - Exon 5	1/5F	5R	126
Exon 1 (120 bp) - Exon 7	1/7F	7R	124
Exon 1 (120 bp) - Exon 8	1/8F	8R	196
Exon 2 - Exon 4	2/4F	5R	222
Exon 2 - Exon 5	2/5F	5R	130
Exon 2 - Exon 8	2/8F	8R	197
Exon 2 - Exon 9	2/9	9R	127
Exon 3 - Exon 5	3/5F	5R	124
Exon 3 - Exon 7	3/7F	7R	127
Exon 3 - Exon 8	3/8F	8R	196
Exon 3 - Exon 9	3/9F	9R	122
Exon 4 - Exon 8	4/8F	8R	197
Exon 4 - Exon 9	4/9F	9R	122
Exon 5 - Exon 7	5/7F	7R	126
Exon 5 - Exon 9	5/9F	9R	123
Exon 6 - Exon 9	6/9F	9R	121
Exon 7 - Exon 9	7/9F	9R	124
Exon N1 - Exon 8	N1/8F	8R	197
Exon 8 - Exon N2	8/N2F	9R	209
