# Supplementary figures and images for: Nanopore Sequencing Unveils Diverse Transcript Variants of the Epithelial Cell-Specific Transcription Factor Elf-3 in Human Malignancies
Source: Genes (Basel). 2021 May 29;12(6):839. doi: 10.3390/genes12060839 (PMC8227732; doi:10.3390/genes12060839)

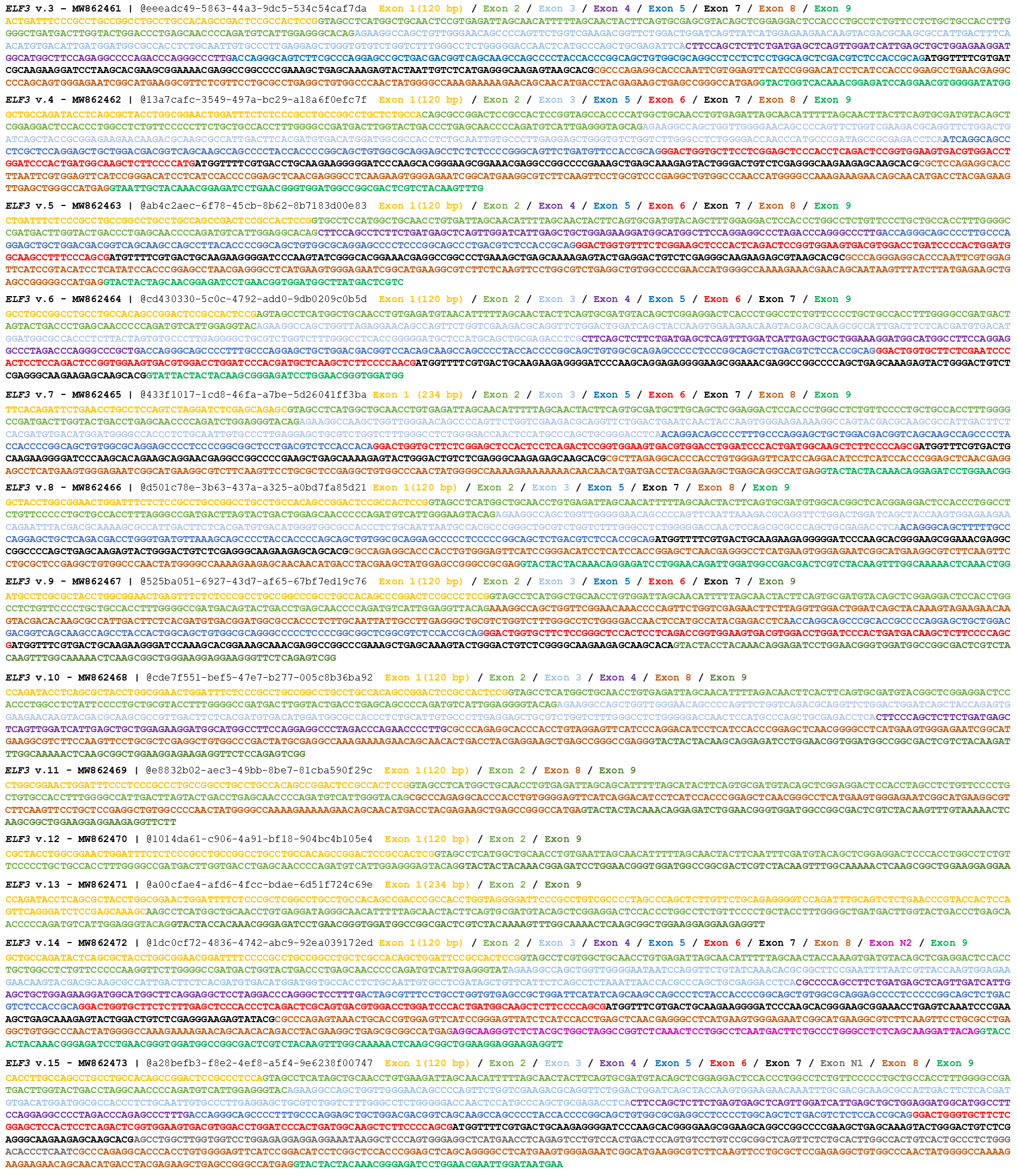

Supplement: Supplementary file 1 [file genes-12-00839-s001.zip › Figure S1.tif]

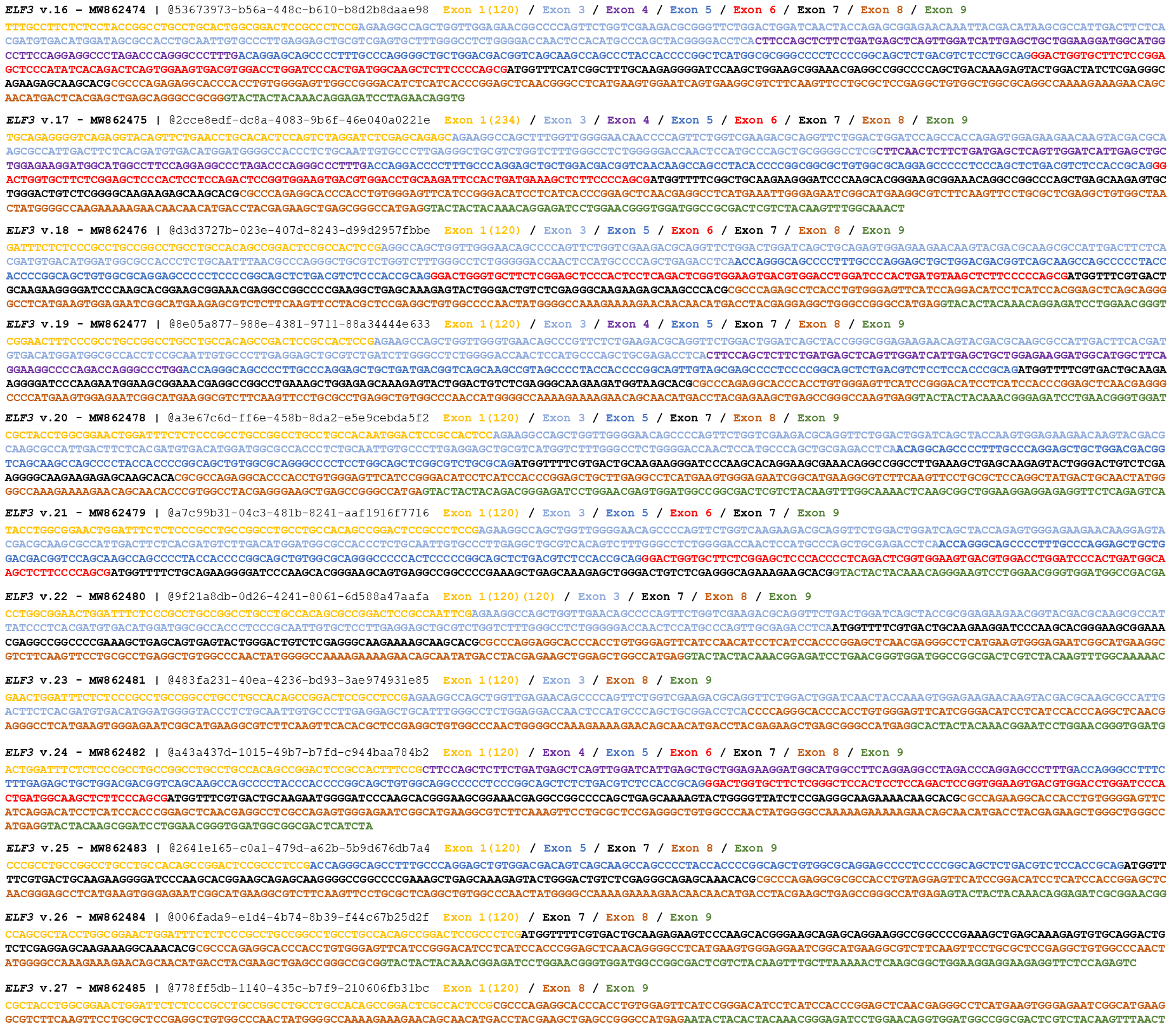

Supplement: Supplementary file 1 [file genes-12-00839-s001.zip › Figure S2.tif]

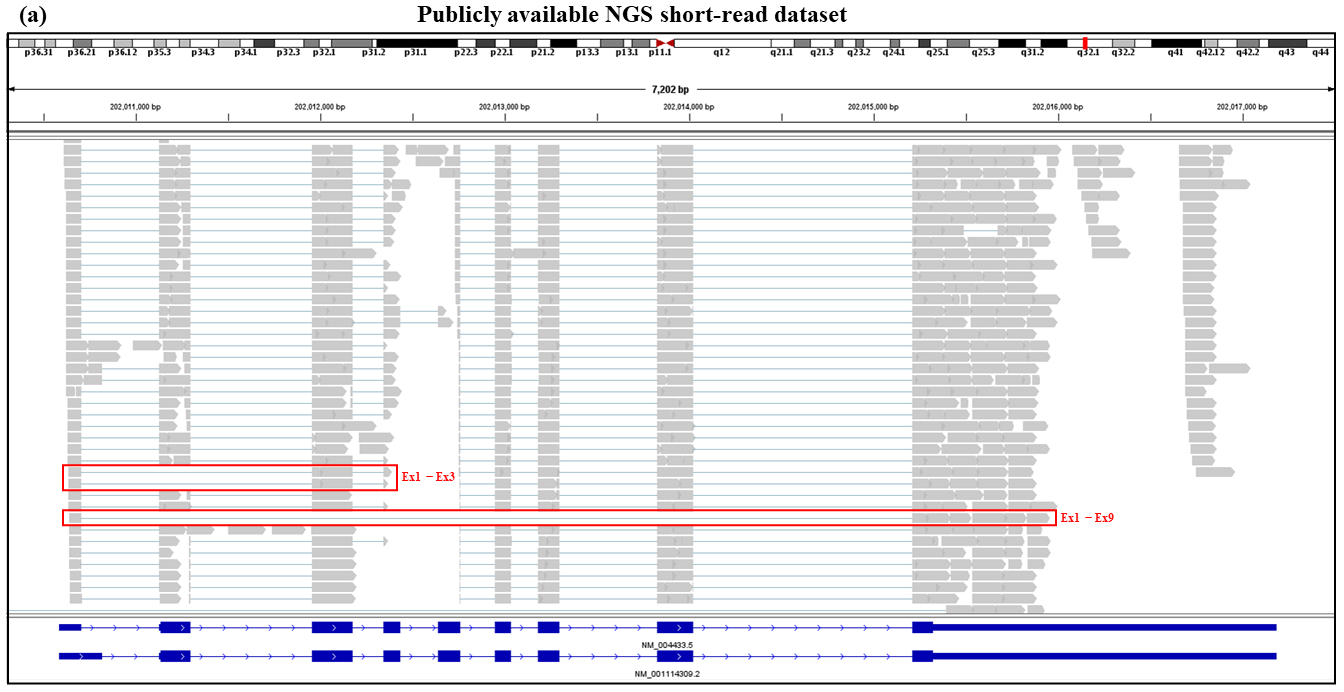

Supplement: Supplementary file 1 [file genes-12-00839-s001.zip › Figure S3a.tif]

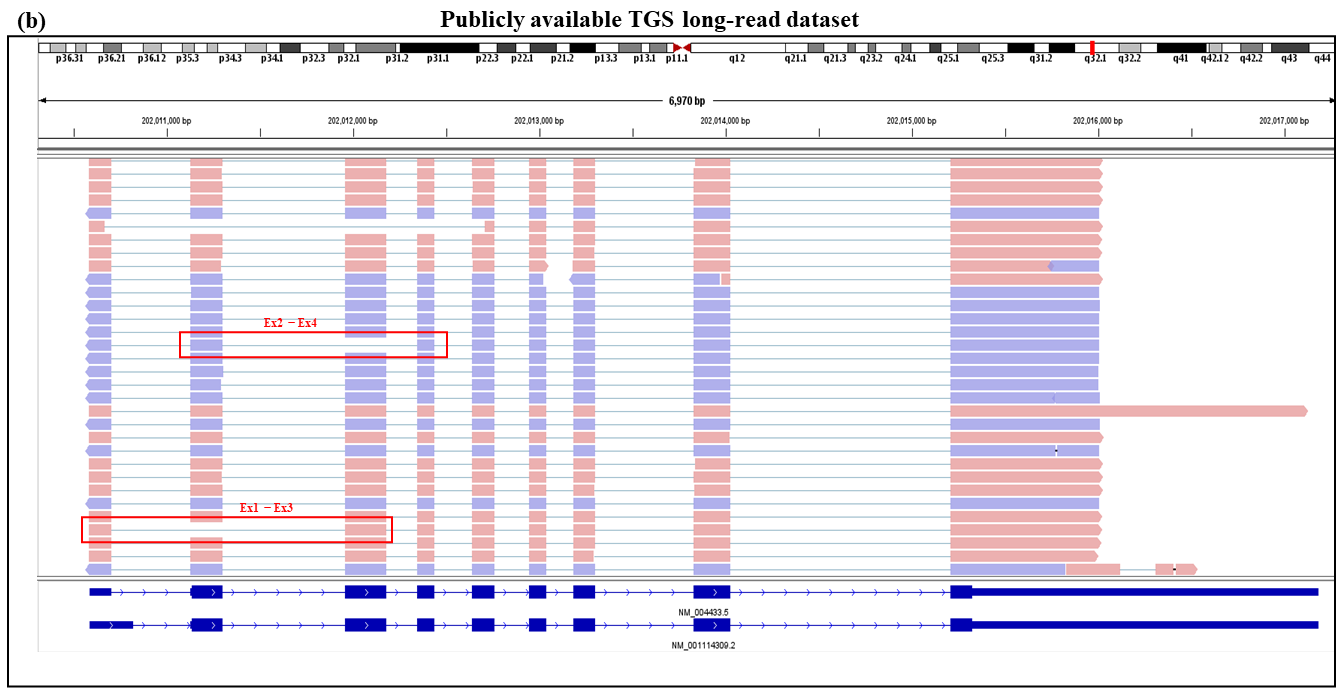

Supplement: Supplementary file 1 [file genes-12-00839-s001.zip › Figure S3b.tif]

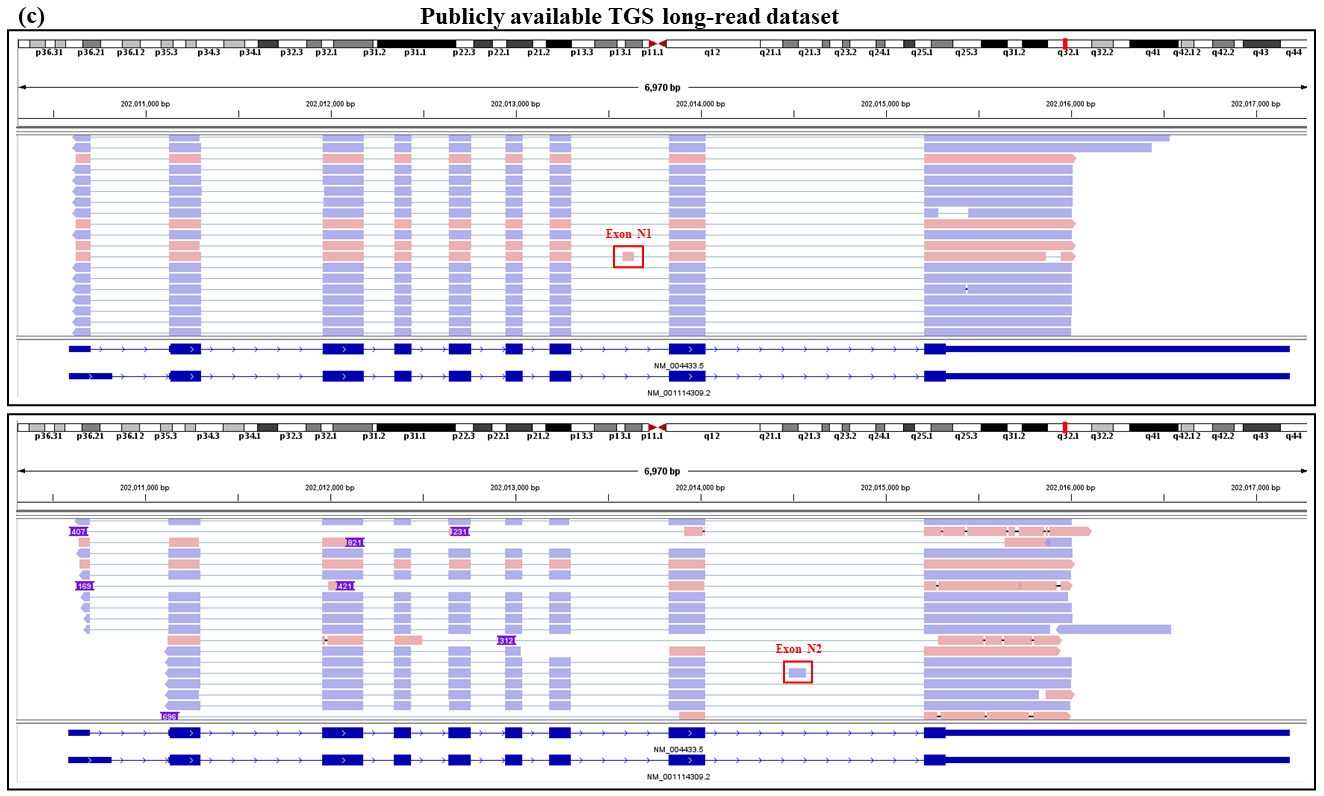

Supplement: Supplementary file 1 [file genes-12-00839-s001.zip › Figure S3c.tif]

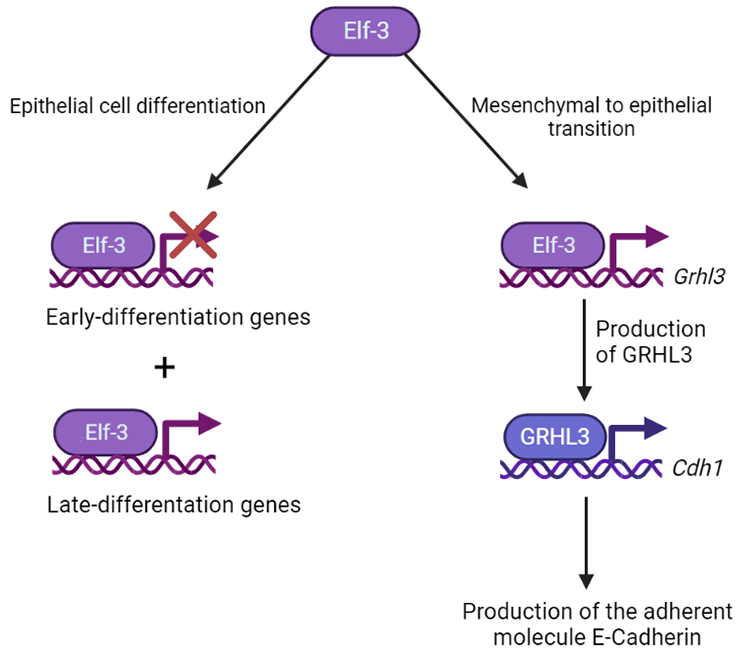

Supplement: Supplementary file 1 [file genes-12-00839-s001.zip › Figure S4.tif]
